# Rhabdomyolysis in a Long-Term Statin User Without Traditional Risk Factors: A Case Report

**DOI:** 10.7759/cureus.46069

**Published:** 2023-09-27

**Authors:** Hiroyuki Naritaka, Yoshitaka Aoki, Yukako Obata, Soichiro Mimuro, Yoshiki Nakajima

**Affiliations:** 1 Department of Anesthesiology and Intensive Care Medicine, Hamamatsu University School of Medicine, Hamamatsu, JPN

**Keywords:** drug interaction, creatine kinase, atorvastatin, adverse event, rhabdomyolysis, statins

## Abstract

We report a rare case of rhabdomyolysis in a 64-year-old man who had been receiving long-term statin therapy for hyperlipidemia. The patient initially presented with symptoms of acute appendicitis, which later progressed to acute renal failure and rhabdomyolysis. No commonly identified risk factors for rhabdomyolysis, including drug interactions and statin doses, were observed. The patient was urgently admitted to the intensive care unit where the relevant medications were discontinued in a timely manner and infusion resuscitation was performed. Renal function and serum creatine kinase levels gradually stabilized without the need for hemodialysis. After four days, the patient was transferred to a general ward and was fully discharged from the hospital 13 days after admission. This case highlights the importance of considering rhabdomyolysis as a possible complication among patients receiving statin therapy, even in the absence of traditional risk factors.

## Introduction

Rhabdomyolysis is a distinct clinical syndrome characterized by necrosis of skeletal muscle cells, which results in a pronounced elevation in serum creatine kinase (CK) concentrations. This pathological condition manifests as a spectrum of clinical presentations, ranging from muscular weakness and myalgia to myoglobinuria, and may escalate into life-threatening complications in severe cases. The incidence of statin-induced rhabdomyolysis is approximately 0.1%. However, the risk is significantly elevated with concomitant drug interactions and high-dose statin therapy [1-3]. Herein, we describe an atypical case of rhabdomyolysis following protracted statin administration that diverged from conventionally documented presentations.

## Case presentation

A 64-year-old Japanese man presented to our emergency department with complaints of right lower abdominal discomfort, diarrhea, emesis, and loss of appetite for two days. A positive McBurney’s sign supported the computed tomography (CT)-based diagnosis of appendicitis. Medical history revealed atrial fibrillation, arterial hypertension, and hyperlipidemia. The patient had been consistently taking atorvastatin (10 mg), azilsartan (40 mg), and edoxaban (60 mg) daily for a decade. Additionally, for the past six years, he had been administered cilnidipine (20 mg), bisoprolol (5 mg), and pilsicainide (150 mg) daily. He denied any recent changes to his pharmacological regimen.

The patient also reported bilateral upper arm muscular discomfort and mentioned a fall in his bathroom one day prior to admission, which was followed by a prolonged period of immobilization due to muscle weakness. Biochemical analysis revealed elevated creatinine levels, suggesting acute kidney injury (AKI), and elevated levels of aspartate, transaminase, and alanine transaminase, indicating impaired liver function. His serum CK level was 64,824 U/L. Renal and hepatic ultrasonography and contrast-enhanced CT did not show any abnormalities. Urinalysis revealed occult hematuria, but no erythrocytes. These clinical parameters led to a supplementary diagnosis of rhabdomyolysis. In this patient, etiological factors such as myofibrillar injury, perturbations in tissue hemodynamics, toxicological agents, endocrinological imbalances, and thermoregulatory abnormalities were ruled out as causative agents for rhabdomyolysis. Consequently, the diagnosis of atorvastatin-induced rhabdomyolysis was established through a process of exclusion. Upon initial evaluation, the patient was fully conscious and had the following vital parameters: height = 170.0 cm; weight = 88.3 kg; body mass index = 30.6 kg/m2; core temperature = 37.8°C; arterial pressure = 127/74 mmHg; cardiac rate = 75 beats/minute; and oxygen saturation = 98% under a 3 L/minute nasal oxygen supply. Pertinent laboratory data and medication history are presented in Table 1.

**Table 1:** Laboratory findings on hospital admission and preadmission medication profiles. BUN, blood urea nitrogen; eGFR, estimated glomerular filtration rate; AST, aspartate aminotransferase; ALT, alanine transaminase; ALP, alkaline phosphatase; CRP, C-reactive protein; CK, creatine kinase; WBC, white blood cells; RBC, red blood cells; MCV, mean corpuscular volume; MCH, mean corpuscular hemoglobin; MCHC, mean corpuscular hemoglobin concentration; PT, prothrombin time; INR, international normalized ratio; APTT, activated partial thromboplastin time; OD, once daily; pCO2, partial pressure of carbon dioxide.

Metabolic panel		Complete blood picture		Home medication
Sodium (Na)	142 mmol/L	WBC	15,300 /uL	Atorvastatin 10 mg OD
Potassium (K)	4.1 mmol/L	RBC	492 x10^4 /uL	Azilsartan 40 mg OD
Chloride (Cl)	104 mmol/L	Hemoglobin	14.6 g/dL	Edoxaban 60 mg OD
pCO_2_	39.2 mmHg	Hematocrit	45.6%	Cilnidipine 20 mg OD
Anion gap	13.0 mmol/L	MCV	93 fL	Bisoprolol 5 mg OD
Glucose	121 mg/dL	MCH	29.7 pg	Pilsicainide 150 mg OD
BUN	60.2 mg/dL	MCHC	32.0 g/dL	
Creatinine	4.78 mg/dL	Platelets	13.9 x10^4/uL	
BUN/creatinine	12.59	Neutrophils	85.0%	
eGFR	11 mL/min/1.73m2	Lymphocytes	9.5%	
Calcium (Ca)	8.6 mg/dL	Monocytes	5.3%	
Magnesium (Mg)	2.6 mg/dL	Eosinophils	0.0%	
Total bilirubin	2.28 mg/dL	Basophils	0.2%	
AST	463 U/L	Coagulation panel		
ALT	76 U/L	PT	23.2 sec	
ALP	57 U/L	INR	1.94	
Other labs		APTT	41.3 sec	
CRP	36.60 mg/dL			
CK	64824 U/L			

**Figure 1:**
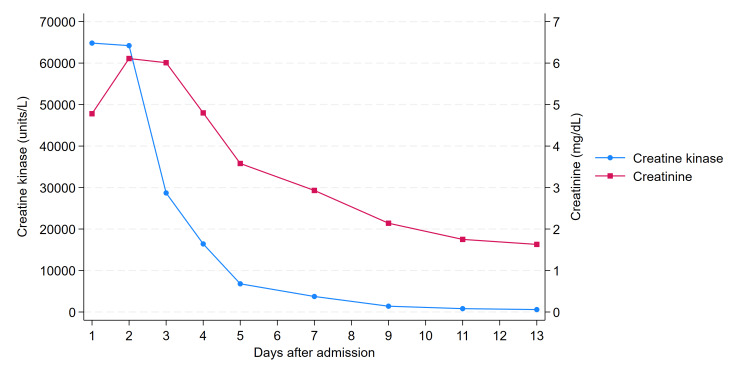
Creatine kinase and creatinine trend

The patient was subsequently transferred to the intensive care unit (ICU) for concurrent management of AKI secondary to rhabdomyolysis and appendicitis. Renal function improved following continuous intravenous fluid resuscitation and sustained diuresis. Serum CK and creatinine concentrations decreased after the cessation of the implicated medications and intravenous hydration (Figure 1). Conservative management strategies, including the administration of antimicrobial agents, were employed to treat the appendicitis. The patient was successfully transferred out of the ICU on the fourth day of hospitalization without requiring hemodialysis and was ultimately discharged home on the 13th day post admission.

## Discussion

We encountered a clinical case of a 64-year-old male patient who developed AKI secondary to rhabdomyolysis following long-term oral administration of low-dose atorvastatin (10 mg). After cessation of statin therapy, the patient exhibited a favorable clinical trajectory; serum CK and creatinine levels progressively declined, enabling successful discharge to a home setting. The constellation of clinical symptoms and laboratory findings did not suggest any alternative pathologies, confirming the diagnosis of atorvastatin-induced rhabdomyolysis.

What distinguishes this case study is the onset of rhabdomyolysis in the absence of multiple risk factors commonly highlighted in the existing literature. A comprehensive analysis of 10,657 cases of rhabdomyolysis, as cataloged in the World Health Organization's VigiBase® pharmacovigilance database, delineates elevated risks associated with simvastatin usage, male gender, advanced age (≥74 years), and specific drug interactions [4]. Network meta-analyses have further substantiated higher incidences of muscle-related complications and elevated CK metrics in high-intensity statin regimens than in moderate-intensity alternatives [5]. In this study, male sex emerged as the sole significant risk factor, whereas low statin dosage and young age were not associated with an increased risk.

Organic anion-transporting polypeptides (OATPs) and hepatic cytochrome P450 3A4 (CYP3A4) have been identified as key mediators of drug interactions involving statins (Table 2) [6]. OATPs are sodium-independent bile-acid transporters expressed on the sinusoidal membranes of human hepatocytes. Novel statins such as atorvastatin, fluvastatin, and cerivastatin are drug substrates of OATP1B1 [7,8]. Hepatic cytochrome CYP3A4 is involved in the metabolism of statins, including their renal and biliary excretion [9]. Cytochrome CYP3A4 inhibitors or competitive substrates inhibit enzyme activity, resulting in decreased statin metabolism, increased plasma statin concentrations, and an increased risk of statin myopathy with new statins [6,10]. Interestingly, neither of these factors was implicated in the clinical course of this patient. Cilnidipine, a dihydropyridine calcium channel blocker that this patient was taking concomitantly, does not fall under the drugs of interaction noted above [11].

**Table 2:** Concomitant drugs reported to interact with statins.

Drug type	Drugs		
CYP3A4 drugs	Allopurinol	Dasatinib	Ketoconazole
	Amiodarone	Delavirdine	Nefazodone
	Amprenavir	Diltiazem	Nifedipine
	Aprepitant	Erythromycin	Quinolones
	Atazanavir	Fluconazole	Ritonavir
	Chloramphenicol	Imatinib	Saquinavir
	Cimetidine	Indinavir	Tamoxifen
	Clarithromycin	Isoniazid	Valproic acid
	Darunavir	Itraconazole	Verapamil
OATP1B1 inhibitors	Atrasentan	Fexofenadine	Rosuvastatin
	Benzylpenicillin	Fluvastatin	SN-38
	Bosentan	Methotrexate	Temocapril
	Caspofungin	Olmesartan	Troglitazone sulfate
	Cerivastatin	Pitavastatin	Valsartan
	Enalapril	Pravastatin	
	Ezetimibe glucuronide	Rifampicin	
Others	Hepatitis C virus direct-acting antivirals	Colchicine	Niacin
	Fibrates	Fusidic acid	

The clinical implications of this case report suggest that rhabdomyolysis should be considered in patients undergoing statin therapy irrespective of the prevalence of associated risk factors. The prescription of statins is escalating due to their well-documented risk-benefit ratio in cardiovascular health [12]. The incidence of rhabdomyolysis is a rare complication that has not shown significant differences even in large randomized controlled trials, but its occurrence may increase as statin prescriptions become more common [13]. However, early diagnosis and appropriate medical intervention can effectively reverse this acute condition. Healthcare practitioners are urged to acquaint themselves with the symptomatic presentations and pertinent laboratory indices associated with this acute ailment [14].

A principal limitation of the present case study was the use of the process of exclusion in the diagnosis of statin-induced rhabdomyolysis. Although we initially posited that inflammation stemming from appendicitis could have been a contributory factor, to the best of our knowledge, no existing literature has corroborated a link between appendicitis and rhabdomyolysis. The potential contributory role of appendicitis-induced inflammation remains inconclusive, given that instances of rhabdomyolysis have been reported in the context of inflammatory responses subsequent to hepatic abscesses or SARS-CoV-2 infections [15,16]. Other conventionally acknowledged etiological factors for rhabdomyolysis, such as musculoskeletal trauma, impaired tissue perfusion, and exposure to pharmacological agents or toxic substances, were not pertinent in this case [17].

## Conclusions

We encountered a patient who manifested rhabdomyolysis, notwithstanding the limited risk factors typically correlated with statin-induced rhabdomyolysis. Despite thorough research on statin interactions and long-term use without issues, as seen in this case, specific triggers may still lead to sudden-onset rhabdomyolysis. In individuals undergoing statin therapy, the presence of myalgia, muscle weakness, and increased CK levels should raise clinical suspicion of rhabdomyolysis, regardless of the presence of predisposing risk factors.
